# Two Distinct Genotypes of *Spissistilus festinus* (Say, 1830) (Hemiptera, Membracidae) in the United States Revealed by Phylogenetic and Morphological Analyses

**DOI:** 10.3390/insects11020080

**Published:** 2020-01-23

**Authors:** Elizabeth Cieniewicz, Victoria Poplaski, Melina Brunelli, Jason Dombroskie, Marc Fuchs

**Affiliations:** 1 Section of Plant Pathology and Plant-Microbe Biology, School of Integrative Plant Science, Cornell University, Cornell AgriTech at the New York State Agricultural Experiment Station, Geneva, NY 14456, USA; ecienie@clemson.edu (E.C.); Victoria.Poplaski@bcm.edu (V.P.); mbrunel1@binghamton.edu (M.B.); 2 Department of Plant and Environmental Sciences, College of Agriculture, Forestry, and Life Sciences, Clemson University, Clemson, SC 29634, USA; 3 Interdepartmental Program in Translational Biology and Molecular Medicine, Baylor College of Medicine, Houston, TX 77030, USA; 4 Departments of Chemistry and Biological Sciences, Harpur College of Arts and Sciences, Binghamton University, State University of New York, Binghamton, NY 13902, USA; 5 Department of Entomology, Cornell University, Ithaca, NY 14853, USA; jjd278@cornell.edu

**Keywords:** *Spissistilus festinus*, genetic barcoding, ITS2, mt-COI

## Abstract

*Spissistilus festinus* (Say, 1830) (Hemiptera: Membracidae) is a frequent pest of leguminous crops in the Southern United States, and a vector of grapevine red blotch virus. There is currently no information on the genetic diversity of *S. festinus*. In this study, populations of *S. festinus* were collected in 2015–2017 from various crops and geographic locations in the United States, and fragments of the mitochondrial cytochrome C oxidase 1 (mt-COI) gene and the nuclear internal transcribed spacer 2 (ITS2) region were characterized by polymerase chain reaction and sequencing. Maximum-likelihood and Bayesian analyses of the mt-COI and ITS2 sequences yielded similar phylogenetic tree topologies, revealing two distinct genetic *S. festinus* lineages with all of the specimens from California comprising one phylogenetic clade, alongside a single GenBank entry from Arizona, and all specimens from the Southeastern United States comprising a statistically-supported distinct clade, regardless of host and year of collection. The mt-COI gene fragment showed up to 10.8% genetic distance between the two phylogenetic clades. These results suggest the existence of two genotypes within *S. festinus* in the United States. The only distinct morphological trait between the two genotypes was a less elevated pronotum in the representative specimens from California, compared to the representative specimens from the Southeastern United States. Since this phenotypic feature is inconspicuous, a diagnostic polymerase chain reaction targeting a variable region of the mt-COI fragment was developed to reliably distinguish between the specimens of the two genotypes of *S. festinus* and to facilitate their specific identification.

## 1. Introduction

The three-cornered alfalfa hopper, *Spissistilus festinus* (Say, 1830) (Hemiptera: Membracidae), is an agricultural pest of legume crops, in particular peanuts, soybeans, and alfalfa in the Southern United States [1]. Recently, the capacity of *S. festinus* to transmit the grapevine red blotch virus (GRBV) from infected to healthy grapevines in the greenhouse was reported [2]. *Spissistilus festinus* is also associated with the red blotch disease spread in vineyards in California [3,4]. *Spissistilus festinus* is not considered to be a direct pest of *Vitis* spp. although it can cause girdling damage of the shoots and petioles upon feeding. Females oviposite in green grapevine tissue but on grapevines, the progeny do not survive to adulthood [5]. No information is available on the genetic relationship between *S. festinus* populations from the Southern United States and California.

Molecular markers have been widely used to determine phylogenetic relationships among insects [6,7,8,9]. For subspecies phylogenetic characterization, several markers have been applied but the mitochondrial cytochrome C oxidase (mt-COI) “barcode” region is the most widely used [10]. For example, mt-COI is routinely used to resolve sub-species phylogenetic relationships and cryptic species identification within *Bemisia tabaci* (Gennadius, 1899) (Hemiptera: Aleyrodidae), one of the most agriculturally important hemipteran pests globally [11,12,13]. Awareness of subspecies variation and cryptic species is important because *B. tabaci* biotypes might have different virus transmission efficiencies [14,15,16] and susceptibility to insecticides [17,18,19].

As *S. festinus* is a long-known pest of leguminous crops in the Southern United States and a recently identified problem for grape production as a vector of GRBV in California, understanding the population structure of this treehopper is important to fill a gap of knowledge and better inform the management of this pest in agriculture production systems, particularly in vineyards. In this study, we investigated the genetic diversity of *S. festinus* populations collected over multiple years, geographic locations, and from various crops. We examined the genetic variation of a 547-nt ‘code’ region of mt-COI, as well as a 445-nt nuclear internal transcribed spacer 2 (ITS2) region of the representative specimens of each *S. festinus* population. Genetic information was combined with morphological analyses to identify two genotypes of *S. festinus* in the United States.

## 2. Materials and Methods

### 2.1. Spissistilus festinus Specimen Collection

*Spissistilus festinus* adults were collected from various crops (clover, alfalfa, soybean, peanut, grape, and weeds) in multiple states in 2015, 2016, and 2017 (Table 1). At each site, between 12 and 25 *S. festinus* were captured. *Spissistilus festinus* adults collected from *Vitis vinifera* ‘Cabernet franc’ in Napa County, California were collected on yellow sticky cards in June and July of 2015 and 2016 [3]. All remaining specimens were collected by sweep netting. Specimens from Alabama, Mississippi, Georgia, North Carolina, and Virginia were preserved in 100% ethanol, upon collection. Ethanol was drained for shipment to the laboratory at Cornell University in Geneva, New York for genotyping, and specimens were then stored at −20 °C, until nucleic acid extraction. Specimens from Parlier, Lodi, Knights Landing, and Davis in California were immediately shipped following collection and stored at −20 °C, until nucleic acid extraction.

### 2.2. Morphological Characteristics of Spissistilus festinus

Adult and nymph specimens of *S. festinus* from Lodi, California and Auburn, Alabama were analyzed by taking into account phenotypic descriptions that were previously published [20]. The genitalia of the specimens of the two states were dissected and observed under a stereoscope. Collection vouchers at the Cornell University and the Smithsonian Institute were used as reference for comparative morphological characteristics. Specimens were photographed with a Macroscopic Solutions Pro and Canon EOS 6D DSLR camera body using the Macro Photo MP-E 65 mm f/2.8 1-5X manual focus lens for EOS, while genitalia were photographed in ethanol on a glass microscope slide, under a cover slip with an EF 70–200 mm zoom lens with a 10X Mitutoyo objective lens.

### 2.3. DNA Extraction, PCR Amplification, and Sequencing of Spissistilus festinus DNA Fragments

Total DNA was extracted from individual specimens using the E.Z.N.A Insect DNA kit (OMEGA Biotek) and DNA was stored at −20 °C for polymerase chain reaction (PCR), using specific primers. Primers ‘LCO1490’ and ‘HCO1298’ were used to amplify a 710-bp fragment from the mitochondrial cytochrome C oxidase subunit 1 gene (mt-COI) [21] by PCR. HotStarTaq polymerase (Qiagen) was used for all PCRs at manufacturer-suggested conditions, with an annealing temperature of 54 °C. Unexpectedly, the mt-COI barcode region was amplified with these primers only for the *S. festinus* populations from California. No mt-COI amplicon was obtained for the *S. festinus* populations from Alabama, Mississippi, Georgia, North Carolina, and Virginia.

To recover the mt-COI barcode region of *S. festinus* populations from the Southeastern United States, tRNA-based primers ‘tRWF2_t1’ and ‘tRWF2_t1’ (both containing the universal primer sequence of ‘M13F’ at the 5′ end) were used in a cocktail as forward primers, in combination with the alternative universal reverse primer ‘LepR1’, to amplify a larger region and recover the region in which the HCO1298/LCO1490 primers failed [22]. A specimen from the Alabama collection was used for this assay. The resulting 900-bp amplicon was directly Sanger-sequenced at the Cornell University Biotechnology Resource Center (Ithaca, NY, USA) using primers ‘M13F’ and ‘LepR1’. Based on a 900-bp sequence fragment, a forward primer (‘SETCAHfor’) was then designed to anneal at the same region as the ‘LCO1490’ primer to amplify a fragment of the mt-COI barcode region of *S. festinus* populations from the Southeastern United States, using PCR. For these *S. festinus* specimens, primers ‘SETCAHfor’ (5′-TTTCTACAAGCCACAGGGATATTGG-3′) and ‘LepR1’ [23] were used to amplify a 710 bp mt-COI barcode fragment. PCR products were resolved by electrophoresis on 2% agarose gels and staining with GelRED (Biotium, Fremont, CA 94538, USA), and was directly Sanger-sequenced at the Cornell University Biotechnology Resource Center (Ithaca, NY, USA), using the PCR primers.

The internal transcribed spacer 2 (ITS2), a non-coding region located between the genes encoding the 5.8S and 28S ribosome subunit, was also amplified by PCR and sequenced. Primers ‘Cas5p8Fc’ and ‘Cas28b1d’ were used to amplify a 500 bp fragment containing the ITS2 region [24], through PCR. DNA amplicons were resolved using electrophoresis (as described above) and directly Sanger-sequenced at the Cornell University Biotechnology Resource Center (Ithaca, NY, USA), using the PCR primers.

### 2.4. Sequence Analyses and Phylogenetic Analyses

PCR products of the *S. festinus* mt-COI and ITS2 genomic regions were purified using ExoSAP-IT (Applied Biosystems, Foster City, CA, USA) and directly sequenced in both directions, using PCR primers. Sequences of mt-COI were obtained from 8 to 24 specimens per *S. festinus* population, depending on the number collected for each population. ITS2 sequences were obtained for at least two specimens from each *S. festinus* population. Sequences from each population were aligned, primer sequences were removed, and consensus sequences were built. Sequences determined in this study were assembled and manually edited using the Lasergene software suite (Version 15.0), aligned using MUSCLE [25], and phylogenies were constructed using RaxML [26] and MrBayes [27]. Branching confidence was estimated using 1000 bootstrap replicates for Maximum Likelihood analyses. Genetic distance matrices were computed in MEGA version X, using the number of pairwise differences per site (p-distance method) [28]. Based on the overall quality of sequences, 547 nucleotides were considered in the mt-COI multi-alignment and phylogenetic analyses. The mt-COI phylogeny was constructed using the sequences determined in this study and GenBank mt-COI sequence entries of at least 500 bp in length. Similarly, ITS2 consensus sequences were aligned and a phylogeny was constructed with 445 nucleotides considered. No other ITS2 sequences of Membracidae were included in our analysis because none were found in GenBank. To assess within-population variability of the mt-COI sequences, a pairwise identity matrix based on a MUSCLE alignment was constructed using the Sequence Demarcation Tool (Version 1.2) [29]. ITS2 stemloop predictions were confirmed using the ITS2 Database [30]. Representative mt-COI and ITS2 sequences for each population were submitted to the NCBI GenBank.

### 2.5. Diagnostic PCR for Distinction of Spissistilus festinus Genotype

Based on highly variable regions of the mt-COI nucleotide alignment between the two *S. festinus* genotypes, PCR primers were designed for the specific detection of each genotype. Primers designed to amplify a 496 bp fragment of the mt-COI gene of the California genotype of *S. festinus* in PCR were ‘TCAHcoiWestF’ (5′-GAATTGGGACAACCAGGACC-3′) and ‘TCAHcoiWestR’ (5′-AACTGGAAGAGACATGAGG-3′). Primers designed for the Southeastern United States genotype of *S. festinus* were ‘TCAHcoiEastF’ (5′-CCTCCGTCTATAATTCTACTCCTTA-3′) and ‘TCAHcoiEastR’ (5′-CCTGCGTAAGTGTAGGGAGAAAATGGCG-3′), to amplify a 145 bp region in PCR. These primers were combined for 12.5 µL PCR reactions, with the following reaction setup: 2.5 µL of 10X PCR Buffer (Qiagen), 1.0 µL of each primer at 10 µM, 0.25 µL of dNTP mix (10 mM each nucleotide) (Thermo Scientific), 0.125 µL of HotStar Taq polymerase (Qiagen), and 4.625 µL of nuclease-free water. PCRs were run at the following temperature cycling protocol: 95 °C for 5 min; 30 cycles of 94 °C for 30 s, 59 °C for 60 s, 72 °C for 60 s; and 72 °C for 10 min. PCR products were resolved by gel electrophoresis on 2% agarose gels, stained with GelRED (Biotium) and imaged under UV light.

The sensitivity and specificity of the diagnostic PCR was validated by testing for cross-amplification of the *S. festinus* genotypes and for amplification of the mt-C2OI gene fragment from other hemipteran insects, respectively. The DNA extracts of various hemiptera were available in a laboratory collection at Cornell University from previous insect survey studies in California and in New York vineyards [3,4].

## 3. Results

### 3.1. Two Distinct Spissistilus festinus Genotypes Based on Geography

**Figure 1:**
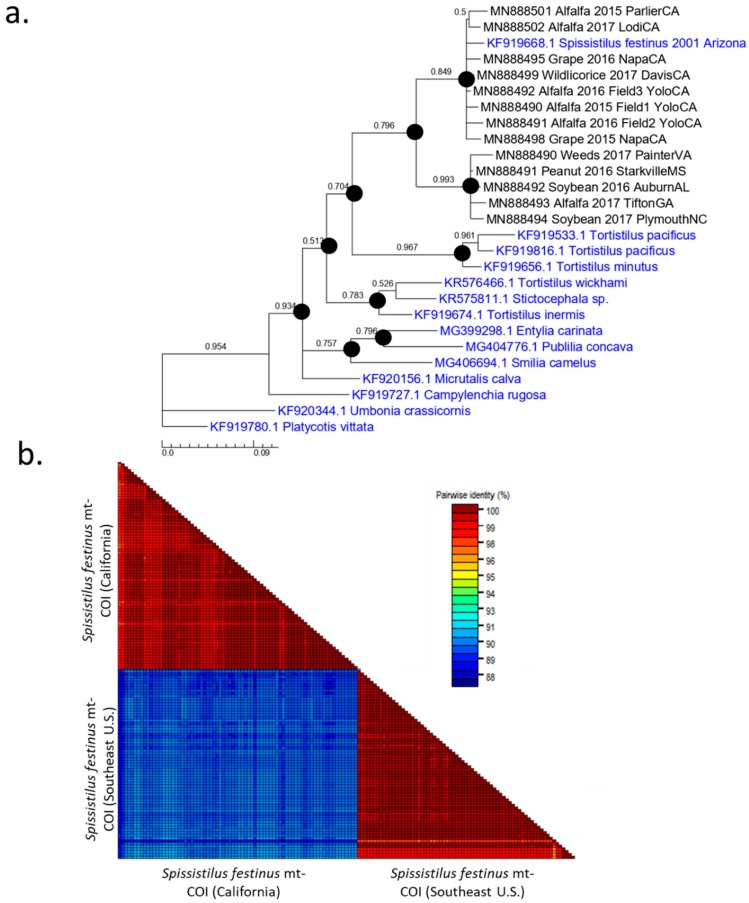
Analyses of mitochondrial cytochrome C oxidase I (mt-COI) consensus sequences (547 nucleotides) from *Spissistilus festinus* populations collected from various locations, years, and crops in California and the Southeastern United States. (**a**) Bayesian inferential analysis indicating two distinct genotypes based on geographic location. Membracid mt-COI sequences determined in this study are denoted in black; the sequences retrieved from GenBank and used for comparison are denoted in blue. Numbers at the nodes indicate Bayesian posterior probability. Nodes denoted with a black dot indicate support by Maximum Likelihood analysis with at least 90% bootstrap support (1000 bootstrap replicates). (**b**) Pairwise identity matrix based on MUSCLE alignment of sequences from individual specimens. Each square depicts a pairwise comparison, where red indicates high sequence identity (>98%) and blue indicates lower (88%–90%) sequence identity. Note the high homogeneity among sequences within populations, and a distinction between mt-COI sequences from *S. festinus* specimens from California and the Southeastern United States.

A partial mt-COI DNA fragment from populations of *S. festinus* collected in 2015–2017, from several states and different hosts, was analyzed through PCR and sequencing. A population of *S. festinus* consisted of specimens from a given host, location, and year. The sequences of mt-COI fragments determined in this study were submitted to GenBank as accession numbers MN888490-MN888502. All populations of *S. festinus* had low within-population nucleotide sequence variation (between 0% and 1%) within the 547 bp sequenced mt-COI genomic region. Phylogenetic analyses of mt-COI sequences revealed two distinct clades (Figure 1a). Populations from California formed one clade, with a subclade containing specimens from Central California (Lodi and Parlier), and another subclade consisting of specimens from Northern California (Davis, Knights Landing, and Rutherford) (Figure 1a). The California populations were otherwise indistinguishable from each other, regardless of source crop or time of collection. The populations from the Southeastern United States (Alabama, Georgia, Mississippi, North Carolina, Virginia) also formed one clade and were indistinguishable from each other. The genetic distance within the “California” clade ranged from 0.0%–0.9%, and 0.0%–0.2% in the “Southeastern United States” clade (Table S1). Genetic distance between these two clades ranged from 10.1%–10.8%, within the 547 nucleotides considered (Figure 1). Interestingly, a single *S. festinus* GenBank mt-COI sequence entry from Arizona (accession number KF919668) grouped with the specimens from California. The existence of two phylogenetic clades of *S. festinus* was confirmed by a pairwise identity matrix of mt-COI sequences from individual specimens (Figure 1b).

**Figure 2:**
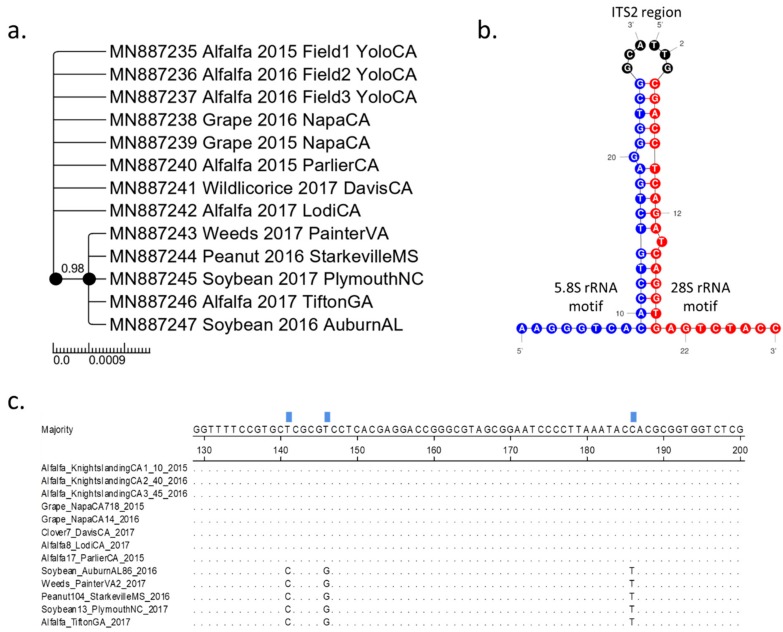
Analyses of internal transcribed spacer 2 (ITS2) sequences (495 nucleotides) from *Spissistilus festinus* populations collected from various locations, years, and crops in California and the Southeastern United States. (**a**) Phylogeny produced by Bayesian inferential analysis (with posterior probability indicated at the node) with the crop, location, and year of collection indicated in the node names. Black dots at nodes indicate bootstrap support of at least 80%, through Maximum Likelihood analysis (1000 bootstrap replicates). (**b**) Depiction of 5.8S (blue) and 28S (red) motifs denoting the boundaries of the ITS2 region (black) used as a nuclear marker for genetic variation among populations of *S. festinus*. (**c**) Segment of MUSCLE alignment of ITS2 sequences in which three nucleotide differences at positions 141, 146, and 186 were observed between *S. festinus* specimens from California and the Southeastern United States.

The sequences of ITS2 fragments determined in this study were submitted to GenBank as accession numbers MN887235-MN887247. Phylogenetic relationships based on ITS2 sequences were consistent with the grouping of *S. festinus* populations in the two clades observed with the mt-COI sequences. One ITS2 clade had all specimens from California and the other clade had all specimens from the Southeastern United States (Figure 2a). For all *S. festinus* ITS2 sequences, the predicted 5.8S and 28S motifs, and the boundaries of the ITS2 region were detected by the annotation function in the ITS2 database (Figure 2b) [30]. This result validated the nature of ITS2 sequences determined in this study. Nonetheless, in comparison to the mt-COI sequences, there was higher nucleotide sequence conservation among the ITS2 sequences (Table S2). In fact, nucleotide differences were only observed at three positions in the 445 nt-long sequence alignment considered within the ITS2 region. *S. festinus* specimens from the Southeastern United States carried a cytosine, a guanine, and a thymine in positions 141, 146, and 186 of the sequenced ITS2 fragment. While *S. festinus* populations from California had a thymine, thymine, and cytosine at the same positions, respectively (Figure 2c). The two distinct clades of ITS2 sequences confirmed the two genotypes of *S. festinus*.

### 3.2. Phenotypic Characterization of the Two Spissistilus festinus Genotypes

**Figure 3:**
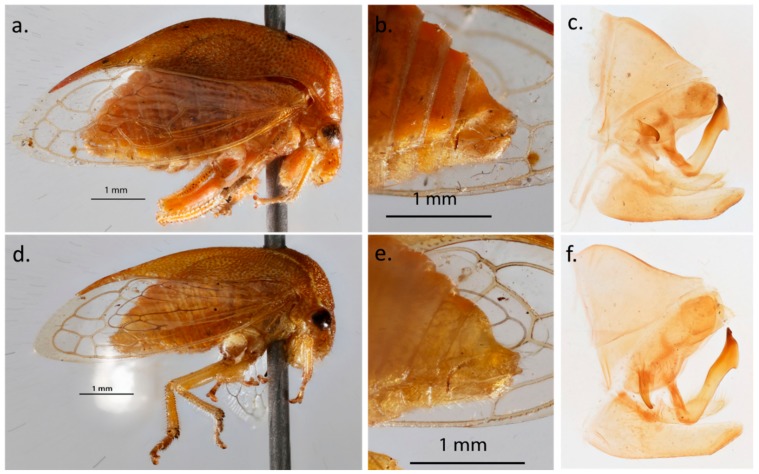
Comparative observations of *Spissistilus festinus* specimens collected from (top panels) soybean in Auburn, Alabama, and (bottom panels) alfalfa in Lodi, California. Full body side view (**a**,**d**), and lateral view of the male terminalia (**b**,**e**) and dissected genitalia (**c**,**f**). Note the less elevated pronotum of the specimen from California compared to the specimen from Alabama.

To verify if the two *S. festinus* genotypes had differential morphological features, specimens from Lodi, California and Auburn, Alabama were analyzed and compared, to each other and to the voucher specimens (Figure 3). These specimens were selected as representatives of the two genotypes identified by phylogenetic analyses. Examination of the terminalia (Figure 3b,e), dissected genitalia (Figure 3c,f), and aedeagus from adult males clearly indicated that specimens from the two geographic origins were *S. festinus*. Indeed, the posterior arm of the aedeagus was tubular in form and longer than the anterior arm, and had a small apical flap above the apically-opening orifice, as previously illustrated [20]. These features are specific to *S. festinus*. Instars from the two *S. festinus* genotypes were 1–3 mm in length with a series of dorsal spine-like protrusions. Adults from the two *S. festinus* genotypes had typical triangular-shaped bodies of 5–7 mm in length, with an elongated pronotum and clear wings (Figure 3a,d). Suprahumeral horns were very prominent and the pronotum was low, compared to other membracids [20]. Interestingly, specimens from California had a less elevated pronotum, especially medially in lateral view, than specimens from Alabama (Figure 3a,d). This was the only obvious morphological trait that distinguished representative specimens from the two *S. festinus* genotypes.

### 3.3. Diagnostic Polymerase Chain Reaction for Spissistilus festinus Genotypes

**Figure 4:**
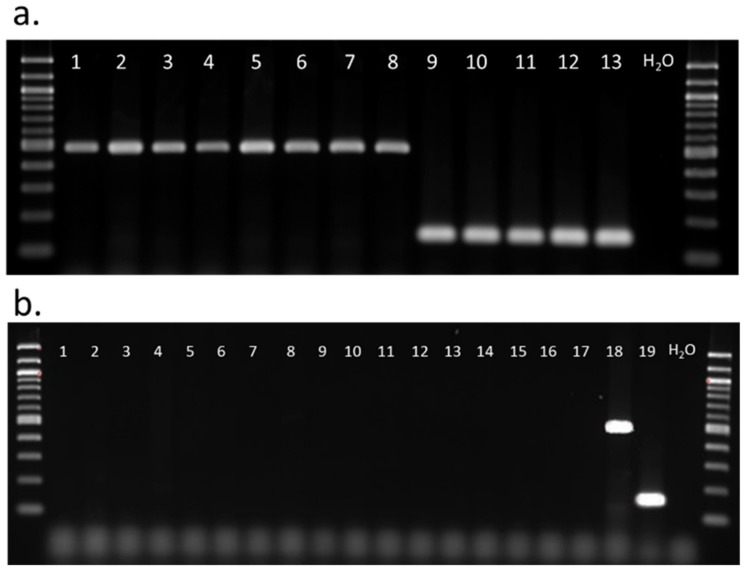
(**a**) Diagnostic polymerase chain reaction for DNA sequence-based identification of specimens of the *S. festinus* genotype from California (lanes 1–8) and the Southeastern United States (lanes 9–13). (**b**) Specificity of the diagnostic PCR against other Membracidae (lanes 1–4), Cercopidae (lanes 5–6), Fulgoroidea (lanes 7–8), Cicadellidae (lanes 9–14), Aphididae (lane 15), Aleyrodidae (lane 16), and Psylloidea (lane 17) compared to the *S. festinus* genotype from California (lane 18) and the Southeastern United States (lane 19).

Since phenotypic differences between the two *S. festinus* genotypes was inconspicuous, a diagnostic PCR assay was designed to distinguish between genotypes of *S. festinus* from California and the Southeastern United States for the amplification of 496 bp and 145 bp DNA fragments, respectively (Figure 4a). The sensitivity of the assay was determined through the specific amplification of DNA targets only from the *S. festinus* specimens (Figure 4a). The specificity of the assay was verified through a lack of amplification of DNA targets from other Membracidae (*Acutalis* sp., *Stictocephala* sp., *Campylenchia* sp., *Entylia carinata*), Cercopidae (*Philaenus* sp., *Clastoptera* sp.), Fulgoroidea (*Delphacidae* sp., *Cixiidae* sp.), Cicadellidae (*Colladonus reductus*, *Osbornellus borealis*, *Scaphytopius* sp., *Empoasca* sp., *Erythroneura elegantula*, *Euscelis* sp.), Aphididae, Aleyrodidae, and Psylloidea (Figure 4b). These results validated the specificity and sensitivity of the diagnostic PCR for the two genotypes of *S. festinus.*

## 4. Discussion

In this study, we collected populations of *Spissistilus festinus* from various geographic locations, crops, and in different years. We analyzed genetic variation within and between populations by sequencing a PCR-amplified fragment of the mt-COI gene and the nuclear noncoding ITS2 region, downstream of the 5.8S rRNA region and upstream of the 28S rRNA region. For both genetic markers there was very little variation within populations, defined as *S. festinus* collections from the same location at the same time. However, based on both genetic markers, two distinct genotypes of *S. festinus* were observed, in which all populations from the Southeastern United States grouped in one clade, and all populations from California grouped into another clade. Notwithstanding, two sub-clades were identified within the mt-COI California clade—one with specimens from Northern California and the other with specimens from Central California.

The mt-COI and ITS2 genetic markers were selected for this study due to their widespread use in insect systematics and availability of degenerate PCR primers for hemipteran insects [9,24,31]. We acknowledge that there might be more accurate genetic markers or methods, such as single nucleotide polymorphism (SNP) datasets for resolving population genetics and phylogeography [6,32]. However, although the ITS2 region displayed very low variability between *S. festinus* genotypes (only three nucleotide substitutions in a 445-nt region), the variability in the ITS2 region did support the mt-COI results and strengthened the identification of two *S. festinus* genotypes. Membracidae speciation based on genomic markers is sparse. Nonetheless, the mt-COI barcode was used for determining subspecies variation in whiteflies [14,15,16,17,18,19] and species variation in planthoppers. Species divergences within tribes Kelisiinae and Asiracinae (Hemiptera: Fulgoroidea) were 12.3% and 13.3%, respectively [33], and 14.4% to 31.1% in species of the genus *Omolicna* [34]. Our observation of 10.1%–10.8% divergence between clades of *S. festinus* supported our hypothesis of the existence of two distinct genotypes.

The two *S. festinus* genotypes were phenotypically very similar, with specimens from California showing a slightly less elevated pronotum than specimens from Alabama. This inconspicuous morphological trait was the only distinct characteristic between the two species. There is currently no known biological difference between *S. festinus* genotypes from California and the Southeastern United States. For example, it is also unknown if these two genotypes are reproductively compatible. Therefore, it is unclear whether the two genotypes described here correspond to two cryptic species. The possible existence of two cryptic species should be evaluated to further resolve the taxonomy of *S. festinus*. Experiments are underway to address this question.

Determining the genotype dispersal in other habitat areas of *S. festinus* that were not surveyed in this study would be of interest to further inform subspecies genetic variation and might shed light on biological dispersal patterns. For example, populations in intermediate localities, such as New Mexico and Texas, warrant such studies to establish which of the two genotypes they are allied to or if they represent additional genotypes. This type of research is justified given the grouping of a single *S. festinus* specimen from Arizona with specimens from California based on nearly identical mt-COI sequences. The diagnostic PCR reported in this study could facilitate *S. festinus* genotype identification and help to refine the actual geographic range of *S. festinus*, as well as areas of co-existence of the two *S. festinus* genotypes.

The geographic range of *S. festinus* is reportedly in the United States, the Caribbean, and in South America [1,35]. However, the southern and northern limits of its range are not well described, and should be further investigated. Noteworthy, although *S. festinus* was not found in a vineyard on Long Island during 2017–2018 insect surveys [4], the Cornell University Insect Collection has a single *S. festinus* specimen collected on Long Island, New York in 1933. Was this specimen migrating from southern habitats before it was caught on Long Island or was it an indigenous specimen? These questions are unanswered, however, it is important to be vigilant for the potential of *S. festinus* to travel north or east, or to be carried in shipments of plant materials, even transiently. Additionally, the geographic range of *S. festinus* might shift in light of climate change and changes in agricultural practices.

Understanding the biological implications of different *S. festinus* genotypes or potential cryptic species, is of interest. Cryptic species with distinct features have been described for diverse insects [9,36,37,38]. For example, one of the cryptic species of the mosquito genus *Aedes* does not carry the gram-negative bacterium *Wolbachia* [39]. Additionally, a different host association is described for a cryptic species of the aphid *Nippolachus piri* [40]. For *S. festinus*, the two genotypes should be evaluated for differences in GRBV transmission capability and efficiency, insecticide resistance, and host range. This is critical to advance our limited understanding of *S. festinus*-GRBV-grapevine interactions and develop optimal pest management tactics. Although *S. festinus* populations appear to be localized toward the edges of vineyards in Napa County, California, primarily reproduce in cover crop species [41], and occur at higher populations at vineyard edges near water sources [3,41], a comprehensive understanding of the *S. festinus* ecology at the landscape level is missing. Such information would be desirable not only to infer landscape-level movements of *S. festinus* through vineyard ecosystems but also to inform pest management recommendations.

## 5. Conclusions

Two distinct genotypes of *Spissistilus festinus* were identified in the United States based on sequence analyses of the mitochondrial cytochrome C oxidase 1 gene and nuclear ITS2 region. Phenotypic information supported the existence of two distinct genotypes of *S. festinus*, with specimens from California having a less elevated pronotum, compared to specimens from the Southeastern United States.

## Figures and Tables

**Table 1:** Sites of *Spissistilus festinus* collection, date of collection, mt-COI primers used for PCR characterization and sequencing, and NCBI GenBank accession numbers.

Location	Latitude	Longitude	Host	Date Collected	Primers for mt-COI PCR and Sequencing	NCBI GenBank Accession Numbers	
						mt-COI Sequences	ITS2 Sequences
Davis, CA	38.5381	−121.8813	Clover and wild licorice	October 2017	HCO1298/LCO1490 ^a^	MN888499	MN887241
Knights Landing, CA	38.7914	−121.7338	Alfalfa	October 2015	HCO1298/LCO1490	MN888490	MN887235
Knights Landing, CA	38.7894	−121.7259	Alfalfa	October 2016	HCO1298/LCO1490	MN888491	MN887236
Knights Landing, CA	38.7660	−121.7911	Alfalfa	October 2016	HCO1298/LCO1490	MN888492	MN887237
Lodi, CA	38.1152	−121.4343	Alfalfa	October 2017	HCO1298/LCO1490	MN888502	MN887242
Rutherford, CA	38.4572	−122.4103	Grape	July 2015	HCO1298/LCO1490	MN888498	MN887239
Rutherford, CA	38.4572	−122.4103	Grape	July 2016	HCO1298/LCO1490	MN888495	MN887238
Parlier, CA	36.5956	−119.5117	Alfalfa	November 2015	HCO1298/LCO1490	MN888501	MN887240
Auburn, AL	32.5927	−85.4858	Soybean	October 2016	SETCAHfor/LepR1 ^b^	MN888492	MN887247
Painter, VA	37.5859	−75.7821	Weeds	July 2017	SETCAHfor/LepR1	MN888490	MN887243
Plymouth, NC	35.6172	−76.7568	Soybean	August 2017	SETCAHfor/LepR1	MN888494	MN887245
Starkville, MS	33.4815	−88.7841	Peanut	October 2016	SETCAHfor/LepR1	MN888491	MN887244
Tifton, GA	31.4887	−83.5413	Alfalfa	August 2017	SETCAHfor/LepR1	MN888493	MN887246

^a^ Primers HCO1298 (5′-TAAACTTCAGGGTGACCAAAAAATCA-3′) and LCO1490 (5′-GGTCAACAAATCATAAAGATATTGG-3′) were developed by Folmer et al. (1994). ^b^ Primer SETCAHfor (5′-TTTCTACAAGCCACAGGGATATTGG-3′) was developed in this study and primer LepR1 (5′-TAAACTTCTGGATGTCCAAAAAATCA-3′) was developed by Hebert et al. (2004).

## Supplementary Materials

The following are available online at https://www.mdpi.com/2075-4450/11/2/80/s1. Table S1: Genetic distance matrix for mitochondrial cytochrome C oxidase I sequences. Pairwise genetic distance (p-distance) is in the lower left corner of the matrix, and standard error is in the top right corner denoted by blue text. Table S2: Genetic distance matrix for internal transcribed spacer 2 sequences. Pairwise genetic distance (p-distance) is in the lower left corner of the matrix, and standard error is in the top right corner denoted by blue text.
