# Supplementary material for: Two Distinct Genotypes of Spissistilus festinus (Say, 1830) (Hemiptera, Membracidae) in the United States Revealed by Phylogenetic and Morphological Analyses
Source: Insects. 2020 Jan 23;11(2):80. doi: 10.3390/insects11020080 (PMC7073536; doi:10.3390/insects11020080)
Supplement: Supplementary file 1 [file insects-11-00080-s001.pdf]

Table S1. Genetic distance matrix for mitochondrial cytochrome C oxidase I sequences. Pairwise genetic distance (p-distance) is in the lower left corner of the matrix, and standard error is in the top right corner denoted by blue text.

			**1**	**2**	**3**	**4**	**5**	**6**	**7**	**8**	**9**	**10**	**11**	**12**	**13**	**14**	**15**	**16**	**17**	**18**	**19**	**20**	**21**	**22**	**23**	**24**	**25**	**26**	**27**
S. festinus Southeast U.S.	1	MN888490	-	0.002	0.002	0.002	0.002	0.013	0.013	0.012	0.012	0.012	0.012	0.012	0.012	0.012	0.016	0.016	0.016	0.016	0.016	0.016	0.018	0.016	0.019	0.017	0.017	0.018	0.017
	2	MN888491	0.002	-	0.000	0.000	0.000	0.013	0.012	0.012	0.012	0.012	0.012	0.012	0.012	0.012	0.016	0.016	0.016	0.016	0.016	0.016	0.017	0.016	0.018	0.017	0.018	0.017	0.017
	3	MN888501	0.002	0.000	-	0.000	0.000	0.013	0.012	0.012	0.012	0.012	0.012	0.012	0.012	0.012	0.016	0.016	0.016	0.016	0.016	0.016	0.017	0.016	0.018	0.017	0.018	0.017	0.017
	4	MN888493	0.002	0.000	0.000	-	0.000	0.013	0.012	0.012	0.012	0.012	0.012	0.012	0.012	0.012	0.016	0.016	0.016	0.016	0.016	0.016	0.017	0.016	0.018	0.017	0.018	0.017	0.016
	5	MN888494	0.002	0.000	0.000	0.000	-	0.013	0.012	0.012	0.012	0.012	0.012	0.012	0.012	0.012	0.016	0.016	0.016	0.016	0.016	0.016	0.017	0.016	0.018	0.017	0.018	0.017	0.016
S. festinus California	6	KF919668	0.106	0.104	0.104	0.104	0.104	-	0.003	0.002	0.002	0.002	0.002	0.002	0.004	0.003	0.017	0.017	0.017	0.017	0.017	0.017	0.017	0.017	0.018	0.019	0.019	0.017	0.016
	7	MN888495	0.106	0.103	0.103	0.103	0.103	0.004	-	0.002	0.002	0.002	0.003	0.003	0.004	0.003	0.016	0.017	0.016	0.016	0.016	0.016	0.016	0.016	0.017	0.018	0.018	0.016	0.016
	8	MN888490	0.104	0.101	0.101	0.101	0.101	0.002	0.002	-	0.000	0.000	0.002	0.002	0.003	0.002	0.017	0.017	0.016	0.016	0.016	0.016	0.016	0.016	0.017	0.018	0.018	0.017	0.016
	9	MN888491	0.104	0.101	0.101	0.101	0.101	0.002	0.002	0.000	-	0.000	0.002	0.002	0.003	0.002	0.017	0.017	0.016	0.016	0.016	0.016	0.016	0.016	0.017	0.018	0.018	0.017	0.017
	10	MN888498	0.104	0.103	0.103	0.103	0.103	0.002	0.002	0.000	0.000	-	0.002	0.002	0.003	0.002	0.017	0.017	0.017	0.016	0.016	0.016	0.016	0.016	0.017	0.018	0.018	0.017	0.017
	11	MN888499	0.106	0.103	0.103	0.103	0.103	0.002	0.003	0.002	0.002	0.002	-	0.000	0.004	0.003	0.017	0.017	0.016	0.016	0.016	0.016	0.016	0.016	0.017	0.018	0.018	0.017	0.016
	12	MN888492	0.106	0.103	0.103	0.103	0.103	0.002	0.003	0.002	0.002	0.002	0.000	-	0.004	0.003	0.017	0.017	0.016	0.016	0.016	0.016	0.016	0.016	0.017	0.018	0.018	0.017	0.016
	13	MN888501	0.108	0.105	0.105	0.105	0.105	0.009	0.008	0.007	0.007	0.007	0.008	0.008	-	0.002	0.017	0.017	0.016	0.016	0.016	0.016	0.016	0.015	0.017	0.018	0.018	0.017	0.017
	14	MN888502	0.104	0.101	0.101	0.101	0.101	0.005	0.005	0.003	0.003	0.003	0.005	0.005	0.003	-	0.017	0.017	0.016	0.016	0.016	0.016	0.016	0.015	0.017	0.018	0.018	0.017	0.016
Other	15	KR576466	0.185	0.182	0.182	0.182	0.182	0.185	0.177	0.179	0.179	0.179	0.181	0.181	0.186	0.182	-	0.002	0.005	0.016	0.016	0.016	0.017	0.016	0.018	0.017	0.018	0.017	0.016

16	KF919674	0.185	0.182	0.182	0.182	0.182	0.187	0.181	0.182	0.182	0.183	0.184	0.184	0.189	0.186	0.003	-	0.005	0.016	0.016	0.016	0.016	0.017	0.018	0.017	0.018	0.018	0.016
17	KF919533	0.186	0.184	0.184	0.184	0.184	0.187	0.177	0.179	0.179	0.179	0.181	0.181	0.186	0.182	0.015	0.015	-	0.016	0.017	0.016	0.017	0.017	0.018	0.017	0.018	0.017	0.016
18	KF919656	0.193	0.191	0.191	0.191	0.191	0.181	0.177	0.176	0.176	0.176	0.177	0.177	0.176	0.176	0.194	0.191	0.189	-	0.013	0.013	0.016	0.015	0.017	0.018	0.018	0.017	0.016
19	KF919816	0.183	0.181	0.181	0.181	0.181	0.192	0.184	0.186	0.186	0.186	0.188	0.188	0.186	0.186	0.181	0.184	0.182	0.111	-	0.012	0.016	0.015	0.017	0.017	0.016	0.016	0.016
20	KR575811	0.179	0.177	0.177	0.177	0.177	0.190	0.188	0.186	0.186	0.186	0.188	0.188	0.182	0.182	0.191	0.194	0.191	0.105	0.105	-	0.016	0.016	0.017	0.017	0.017	0.017	0.016
21	MG399298	0.198	0.196	0.196	0.196	0.196	0.190	0.186	0.184	0.184	0.185	0.186	0.186	0.182	0.182	0.204	0.208	0.203	0.181	0.169	0.188	-	0.014	0.014	0.016	0.017	0.016	0.015
22	MG406694	0.191	0.189	0.189	0.189	0.189	0.183	0.181	0.179	0.179	0.179	0.181	0.181	0.172	0.176	0.204	0.208	0.209	0.193	0.182	0.189	0.137	-	0.014	0.016	0.016	0.016	0.016
23	MG404776	0.222	0.221	0.221	0.221	0.221	0.195	0.193	0.195	0.195	0.194	0.197	0.197	0.191	0.191	0.212	0.216	0.214	0.205	0.190	0.200	0.125	0.139	-	0.018	0.018	0.017	0.017
24	KF919780	0.232	0.230	0.230	0.230	0.230	0.249	0.243	0.242	0.242	0.243	0.242	0.242	0.243	0.240	0.240	0.242	0.236	0.258	0.238	0.245	0.212	0.225	0.239	-	0.016	0.015	0.017
25	KF920344	0.241	0.242	0.242	0.242	0.242	0.253	0.248	0.247	0.247	0.244	0.247	0.247	0.248	0.247	0.258	0.262	0.262	0.245	0.230	0.243	0.230	0.226	0.254	0.190	-	0.015	0.017
26	KF919727	0.222	0.218	0.218	0.218	0.218	0.214	0.211	0.213	0.213	0.215	0.213	0.213	0.211	0.211	0.248	0.250	0.245	0.220	0.201	0.216	0.201	0.213	0.232	0.214	0.223	-	0.016
27	KF920156	0.226	0.225	0.225	0.225	0.225	0.223	0.213	0.211	0.211	0.212	0.213	0.213	0.209	0.209	0.240	0.243	0.236	0.201	0.206	0.209	0.204	0.204	0.230	0.245	0.248	0.226	-
				S. festinus Southeast U.S.							*S. festinus* California										Other Membracidae							

Table S2. Genetic distance matrix for internal transcribed spacer 2 sequences. Pairwise genetic distance (p-distance) is in the lower left corner of the matrix, and standard error is in the top right corner denoted by blue text.

			**1**	**2**	**3**	**4**	**5**	**6**	**7**	**8**	**9**	**10**	**11**	**12**	**13**
S. festinus California	1	MN887241	-	0.00000	0.00000	0.00000	0.00000	0.00000	0.00000	0.00000	0.00367	0.00367	0.00367	0.00367	0.00367
	2	MN887235	0.00000	-	0.00000	0.00000	0.00000	0.00000	0.00000	0.00000	0.00367	0.00367	0.00367	0.00367	0.00367
	3	MN887236	0.00000	0.00000	-	0.00000	0.00000	0.00000	0.00000	0.00000	0.00367	0.00367	0.00367	0.00367	0.00367
	4	MN887237	0.00000	0.00000	0.00000	-	0.00000	0.00000	0.00000	0.00000	0.00367	0.00367	0.00367	0.00367	0.00367
	5	MN887242	0.00000	0.00000	0.00000	0.00000	-	0.00000	0.00000	0.00000	0.00367	0.00367	0.00367	0.00367	0.00367
	6	MN887239	0.00000	0.00000	0.00000	0.00000	0.00000	-	0.00000	0.00000	0.00367	0.00367	0.00367	0.00367	0.00367
	7	MN887238	0.00000	0.00000	0.00000	0.00000	0.00000	0.00000	-	0.00000	0.00367	0.00367	0.00367	0.00367	0.00367
	8	MN887240	0.00000	0.00000	0.00000	0.00000	0.00000	0.00000	0.00000	-	0.00367	0.00367	0.00367	0.00367	0.00367
S. festinus [illegible]	9	MN887247	0.00674	0.00674	0.00674	0.00674	0.00674	0.00674	0.00674	0.00674	-	0.00000	0.00000	0.00000	0.00000
	10	MN887243	0.00674	0.00674	0.00674	0.00674	0.00674	0.00674	0.00674	0.00674	0.00000	-	0.00000	0.00000	0.00000
	11	MN887245	0.00674	0.00674	0.00674	0.00674	0.00674	0.00674	0.00674	0.00674	0.00000	0.00000	-	0.00000	0.00000
	12	MN887244	0.00674	0.00674	0.00674	0.00674	0.00674	0.00674	0.00674	0.00674	0.00000	0.00000	0.00000	-	0.00000
	13	MN887246	0.00674	0.00674	0.00674	0.00674	0.00674	0.00674	0.00674	0.00674	0.00000	0.00000	0.00000	0.00000	-
